# Mediation of transitional B cell maturation in the absence of functional Bruton’s tyrosine kinase

**DOI:** 10.1038/srep46029

**Published:** 2017-04-05

**Authors:** Shalini Tanwar, Atika Dhar, Vineeth Varanasi, Tapas Mukherjee, Ramanamurthy Boppana, Soumen Basak, Vineeta Bal, Anna George, Satyajit Rath

**Affiliations:** 1 National Institute of Immunology, New Delhi, India; 2 National Centre for Cell Sciences, Pune, India

## Abstract

X-linked immune-deficient (Xid) mice, carrying a mutation in Bruton’s tyrosine kinase (Btk), have multiple B cell lineage differentiation defects. We now show that, while Xid mice showed only mild reduction in the frequency of the late transitional (T2) stage of peripheral B cells, the defect became severe when the Xid genotype was combined with either a CD40-null, a TCRbeta-null or an MHC class II (MHCII)-null genotype. Purified Xid T1 and T2 B cells survived poorly *in vitro* compared to wild-type (WT) cells. BAFF rescued WT but not Xid T1 and T2 B cells from death in culture, while CD40 ligation equivalently rescued both. Xid transitional B cells *ex vivo* showed low levels of the p100 protein substrate for non-canonical NF-kappaB signalling. *In vitro*, CD40 ligation induced equivalent activation of the canonical but not of the non-canonical NF-kappaB pathway in Xid and WT T1 and T2 B cells. CD40 ligation efficiently rescued p100-null T1 B cells from neglect-induced death *in vitro*. These data indicate that CD40-mediated signals, likely from CD4 T cells, can mediate peripheral transitional B cell maturation independent of Btk and the non-canonical NF-kappaB pathway, and thus contribute to the understanding of the complexities of peripheral B cell maturation.

Multiple molecular checkpoints regulate the development and differentiation of B lineage cells in the bone marrow and their maturation in the spleen. Dysfunctions of many signalling molecules involved in these checkpoints are known to disrupt B cell development and maturation^1^,^2^. In the bone marrow, the successful formation of the pre-B cell receptor (BCR) and the BCR provide signals for mediating developmental transitions^3^,^4^. Immature BCR-bearing B cells exit the bone marrow as transitional B cells and undergo further maturation in the periphery, possibly in the spleen^5^, via later transitional stages, first to precursors and thence to the mature functional stages of both marginal zone (MZ) and classical follicular B cells^6^,^7^. Peripheral maturation and survival are dependent on constitutive signals from the BCR as well as from the B cell activating factor (BAFF), mostly through one of the three known receptors for BAFF, BAFF-R^8^,^9^,^10^,^11^,^12^. There is evidence for cross-talk between the BCR- and the BAFF-R-mediated signals^9^,^11^, possibly involving feedback loops between the two major arms of the NF-kappaB signalling pathway, namely, the ‘canonical’ one involving p65/p50 and the ‘non-canonical’ one depending on p100-p52/RelB respectively^12^. Crosstalk between these two NF-kappaB pathways is important for transitional and mature B cell survival^13^.

Bruton’s tyrosine kinase (Btk) is a Tec family kinase selectively expressed in the hematopoietic lineage in the myeloid and B lymphocyte compartments^14^, and Btk deficiency in the X-linked immune-deficient (Xid) mouse strain leads to reduction of peripheral mature, particularly follicular B cell numbers^15^,^16^. These data have been interpreted to indicate that BCR signalling is critical for maturation and/or maintenance of peripheral B cells^11^,^17^,^18^,^19^, as has also been shown by more direct manipulations of the BCR^4^,^17^. However, while Btk is clearly involved in signalling from the BCR^3^,^20^, Btk deficiency still allows some BCR-mediated signalling^21^,^22^,^23^,^24^, and Btk is also involved in signalling downstream of other cell-surface molecules on both B and non-B cells^25^,^26^,^27^,^28^,^29^,^30^,^31^. Thus, the roles of BCR- and non-BCR-mediated signals in peripheral B cell maturation, and the significance of Btk in the mediation of those signals are still not well understood.

In this context, previous reports have shown that combining a ‘nude’ Foxn1-null genotype or a CD40-null genotype with the Xid genotype severely exacerbates the Xid phenotype of peripheral B cell scarcity^32^, even though neither the Foxn1-null nor the CD40-null genotype show any B cell deficiency by themselves^33^,^34^. A recent report has also shown that depletion of CD4 T cells in Xid mice led to a maturation block downstream of transitional B cells^35^. Together, these data suggest that there are likely to be mutually redundant mechanisms working in the pathways mediating peripheral B cell maturation. However, the transition from the early (T1) to the late (T2) transitional stage of B cell maturation is a major checkpoint for which regulatory mechanisms are still poorly understood. Thus, while an early report indicated that BCR-mediated signals might be useful primarily from the T2 stage onwards^36^, it is now recognized that signals from the BCR and BAFF-R are integrated for this transition^10^. However, the signal integration indicated by the Xid+ nu/nu or the Xid+ CD40-null double-mutant genotypes has not been examined for its role in this critical transition.

On this background, we have examined the stages of peripheral B cell maturation in Xid mice in comparison to Xid mice also lacking alpha-beta T cells, CD40 or MHCII, as well as the survival and maturation *in vitro* of T1 and T2 B cells from Xid and WT mice. Our data indicate that the T1 to T2 transition in Xid (but not in WT) B cells is dependent on the availability of CD40-mediated signals probably from CD4 T cells, and that these CD40-mediated signals are independent of the non-canonical NF-kappaB pathway, unlike the pathway mediated by cross-talk between the BCR and the BAFF-R. These data provide evidence for redundancies in the pathways mediating peripheral B cell maturation and survival.

## Results

### Xid mice show milder reductions in immature stages than in mature stages of peripheral B cells

Cell numbers of B cell lineage stages in the bone marrow were estimated as previously defined as fraction A (B220^+^CD43^+^CD24^−^ BP1^−^), fraction B (B220^+^CD43^+^CD24^+^BP1^−^), fraction C (B220^+^CD43^+^CD24^int^BP1^+^), fraction C’ (B220^+^CD43^+^CD24^hi^BP1^+^), fraction D (B220^+^CD43^−^IgM^−^), fraction E (B220^+^CD43^−^IgM^+^) and fraction F (B220^hi^CD43^−^IgM^int^) (Supplementary data Fig. S1)^37^. They showed little difference between Xid and WT mice with some modest reductions (Supplementary data Fig. S2), consistent with previous reports^38^. There were also fewer recirculating mature B cells in the Xid bone marrow (Supplementary data Fig. S2), mirroring the peripheral B cell phenotype.

**Figure 1:**
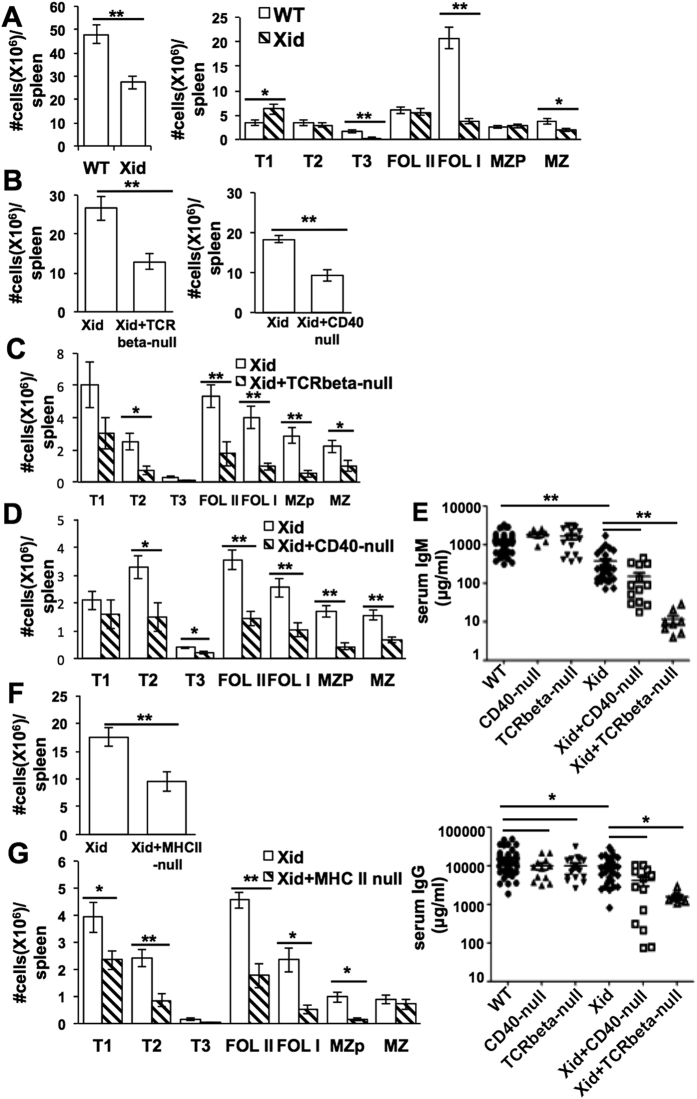
The stage of the peripheral B cell maturation block in Xid mice shifts upstream upon removal of CD4 T cell help. *Ex-vivo* splenic cells from mice of indicated genotypes were stained for B220, CD93, CD23, CD21/35 and IgM to estimate (as shown in Fig. S1), (**A**) numbers of total B cells and of B cell subsets in WT and Xid mice (n = 13), (**B**) numbers of total B cells and (**C,D**) of B cell subsets in Xid versus Xid + TCRbeta-null littermate and Xid versus Xid + CD40-null littermate mice, (n = 9). (**E**) Sera from 4–8 week old littermate mice of various genotypes as shown were analyzed for IgM and IgG levels (n ≥ 5). (**F,G**) *Ex-vivo* splenic cells from mice of indicated genotypes were stained for B220, CD93, CD23, CD21/35 and IgM to estimate numbers of total B cells (**F**) and of B cell subsets (**G**) in Xid versus Xid + MHCII-null littermate mice (as shown in Fig. S1; n = 6). *p < 0.05; **p < 0.005.

However, in a stage-wise analysis of the splenic B cell compartment identifying stages as described as T1 (B220^+^CD93^+^IgM ^hi^CD23^−^), T2 (B220^+^CD93^+^IgM^hi^CD23^+^), T3 (B220^+^CD93^+^IgM^lo^CD23^+^), FolI (B220^+^CD93^–^CD23^+^IgM^int^CD21^int^), FolII (B220^+^CD93^−^CD23^+^IgM^hi^CD21^int^), MZP (B220^+^CD93^−^CD23^+^IgM^hi^CD21^hi^) and MZ (B220^+^CD93^−^CD23^−^IgM^hi^CD21^hi^) (Supplementary data Fig. S1)^39^, it was evident that transitional T1 and T2 stages, as well as the downstream immature follicular (FolII) and marginal zone-precursor (MZP) stages, showed little if any reduction in Xid mice (Fig. 1A). As expected, the reduction in the mature MZ stage was modest, while the most striking reduction was in the mature follicular (FolI) stage (Fig. 1A). Thus, a major defect in peripheral B cell maturation in Xid mice appears to be during the successful transition of immature FolII to mature FolI cells and/or survival of FolI cells.

### Late transitional T2 B cell defect in mice lacking both functional Btk and either alpha-beta T cells, CD40 or MHCII

In the context of the reported major loss of peripheral B cell numbers in Xid+ Foxn1-null and Xid+ CD40-null double-mutant (DM) genotypes^32^,^40^, we examined the B cell lineage stages in Xid+ TCRbeta-null and Xid+ CD40-null DM mice. The background genotypes of the Xid, TCRbeta-null and CD40-null mouse strains were different (see Methods). Since background genotype differences could potentially confound the results, all comparisons were made between groups of F1 × F1 cross-littermate mice with complex but comparable background genotypes. The bone marrow B lineage phenotypes, as expected, by and large showed no differences between the WT and TCRbeta-null mice, or between WT and CD40-null mice (Supplementary data Fig. S2). Similarly, there were by and large no differences between the bone marrow B cell stages of Xid and Xid+ TCRbeta-null DM mice, or between Xid and Xid-CD40-null DM mice (Supplementary data Fig. S2).

However, as compared to Xid mice, the total splenic B lineage cell numbers were much lower in Xid+ TCRbeta-null DM or Xid+ CD40-null DM mice (Fig. 1B). When the peripheral B cell stages were examined, it was apparent that only the T1 B cell stage showed numbers comparable to Xid mice in the Xid+ TCRbeta-null DM or the Xid+ CD40-null DM mice; all subsequent stages of B cell maturation from the T2 stage onwards, including the immature follicular FolII and MZP stages, were reduced in the DM mice compared to Xid mice (Fig. 1C,D). Consistent with these data, we found that both serum IgM and IgG levels were lower in Xid+ TCRbeta-null DM or Xid+ CD40-null DM mice compared to Xid mice (Fig. 1E). While serum IgG levels were significantly lower in CD40-null or TCRbeta-null mice compared to wild-type mice (Fig. 1E), the reduction was modest in these young mice. Older ~8 week-old CD40-null mice showed major reduction in serum IgG levels as expected (Fig. S3). Thus, the addition of a TCR-beta-null or a CD40-null genotype to the Xid genotype leads to an upstream shift in the B cell maturational block.

The ligand for CD40, CD40L, is strongly expressed upon activation on both CD4 and CD8 T cells^41^. Since the role of CD40L-expressing T cells for efficient T1-T2 transition in the Xid genotype would be a non-cognate ‘bystander’ effect, we next asked if either CD4 or CD8 T cells could mediate it. We tested this by generating Xid + MHCII-null DM mice and comparing their T1-T2 transition with that in Xid mice. Again, as compared to Xid mice, the total splenic B lineage cell numbers were much lower in Xid+ MHCII-null DM mice (Fig. 1F). T1 B cell numbers were somewhat reduced, and most subsequent stages from T2 onwards were strikingly reduced in the Xid+ MHCII-null DM mice compared to Xid mice (Fig. 1G), showing that CD4 T cells were essential for T1-T2 transition in the absence of functional Btk.

### While the spleen plays a role in T1-T2 transition in Xid mice, the adhesion molecule Icam-1 is not required

**Figure 2:**
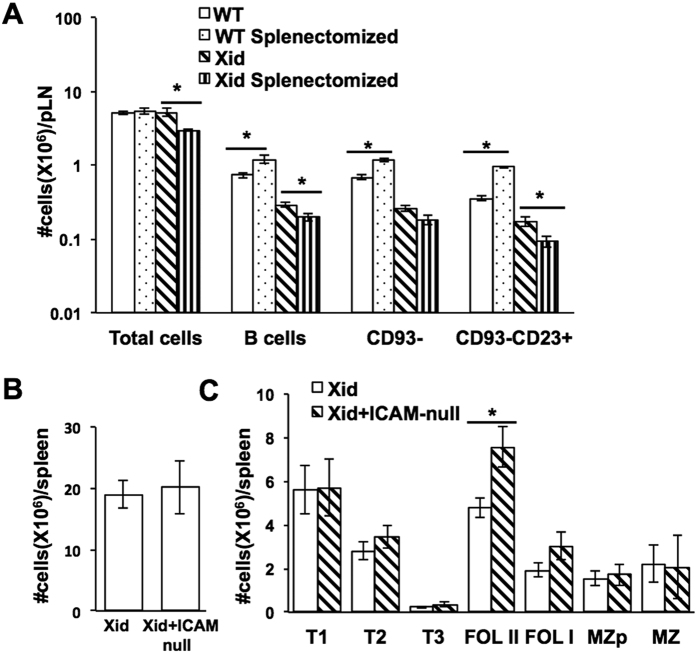
Peripheral B cell maturation in Xid mice in the absence of the spleen or of Icam-1. (**A**) WT or Xid mice were subjected to splenectomy, splenectomised and control mice were gamma-irradiated (5 Gy) ten days later, and lymph node B cell lineage analysis was done 1 month after irradiation. Numbers of total cells, total B lineage cells, and B lineage subsets in the peripheral lymph nodes (pLN) of control and splenectomised WT and Xid mice are shown (n = 4). *p < 0.05. (**B,C**) *Ex-vivo* splenic cells from mice of indicated genotypes were stained for B220, CD93, CD23, CD21/35 and IgM to estimate (as shown in Fig. S1) numbers of (**B**) total B cells and (**C**) B cell subsets in Xid versus Xid + Icam1-null littermate mice (n = 4). *p < 0.05.

The spleen is thought of as a major site for transitional B cell maturation^7^. Since Xid B cells appear to require bystander T cell help for effective maturation, we examined if the spleen was an essential site for such interactions in them. We hypothesized that if the interactions necessary for T1-T2 transition in Xid mice took place only in the spleen, the development of B cells in splenectomised Xid mice would be significantly affected. For this, WT and Xid mice were subjected to total splenectomy and allowed to recover. Subsequently, both control and splenectomised WT and Xid mice were gamma-irradiated with a sub-lethal dose (5Gy) and the bone marrow allowed to undergo spontaneous reconstitution. When peripheral lymph nodes from these mice were examined for B cell numbers, it was evident that, while splenectomy led to an increase in WT B cell numbers, particularly in the more mature B220+ CD93-CD23+ compartment, as reported earlier^42^, it caused a further decrease in these B cell numbers in Xid mice (Fig. 2A), indicating that the spleen is far more crucial for T1-T2 transition of Xid B cells than of WT B cells.

Since B cell trafficking and recirculation through peripheral lymphoid organs depends on interactions of adhesive molecules such as Icam-1^42^, we tested whether lack of Icam-1 would inhibit T1-T2 transition in Xid B cells, using Xid+ Icam1-null DM mice. However, the total splenic B lineage cell numbers were comparable between Xid and Xid+ Icam1-null DM mice (Fig. 2B), as were T1 and T2 stage cell numbers (Fig. 2C).

### Metabolic parameters of transitional B cells are largely unaffected by lack of functional Btk

**Figure 3:**
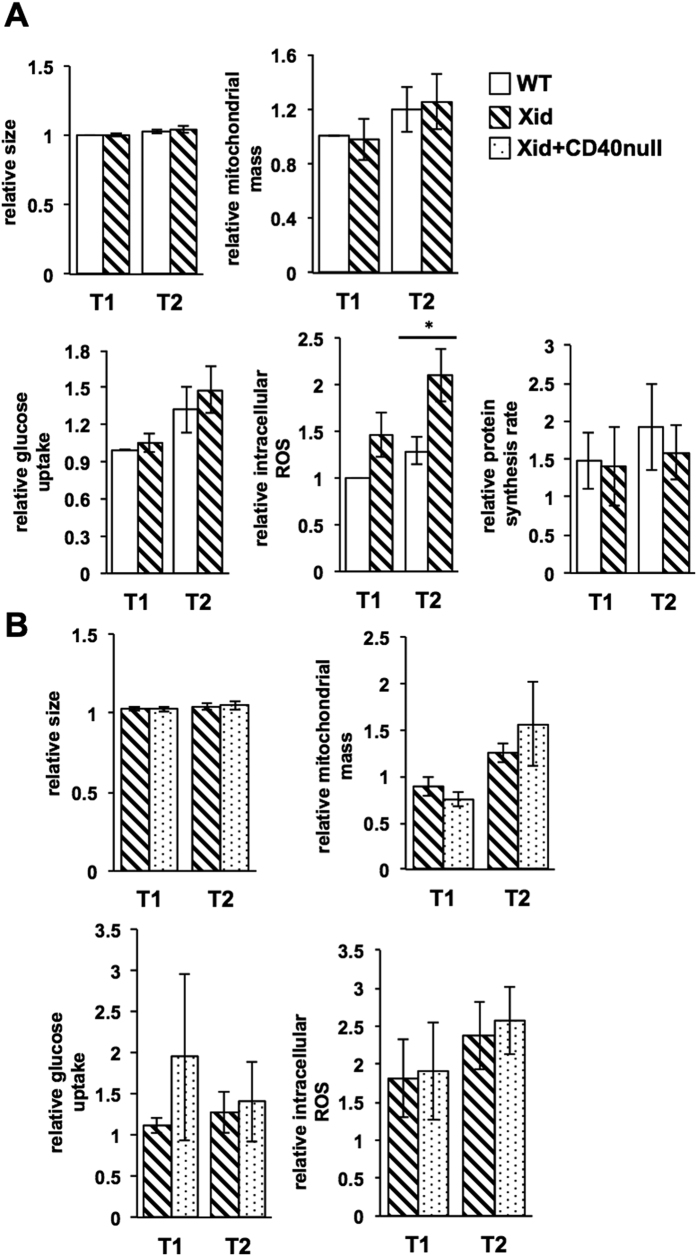
Metabolic parameters of T1 and T2 stage B cells are unaltered in Xid mice. Spleen cells from mice of indicated genotypes were stained as described for the various parameters shown (see Materials and Methods). Parameters were measured as MFI values corrected with reference to unstained cells and normalized to WT T1 (**A**) or Xid T1 (**B**) levels. n = 3–5 independent experiments; *p < 0.05.

Btk is known to function downstream of the BCR and control the PI-3-kinase (PI3K)-Akt pathway^43^, which is a major regulator of cellular metabolism^44^,^45^, likely to be a significant factor in developmental transitions^46^. We therefore examined if parameters reflecting cellular metabolism showed modification during Xid B cell maturation in the periphery. Notably, neither cell size, nor mitochondrial mass, nor uptake of a fluorescent glucose analogue, nor rates of protein synthesis, were different between WT and Xid T1 and T2 transitional B cells (Fig. 3A, Supplementary data Fig. S4). Cellular ROS levels did show modest but consistent differences between WT and Xid T2 B cells (Fig. 3A). However, cellular ROS levels did not show any differences between Xid and Xid+ CD40-null T1 or T2 B cells (Fig. 3B), suggesting that they were unlikely to be related to the enhanced loss of T2 B cells seen in the DM mice. Similarly, neither cell size, nor mitochondrial mass, nor uptake of a fluorescent glucose analogue, were different between Xid and Xid+ CD40-null T1 or T2 B cells either (Fig. 3B). Together, these data indicate that the metabolic status of peripheral B lineage cells may be largely independent of both Btk and CD40 signals.

### CD40 ligation rescues neglect-induced death in Xid transitional B cells, while BAFF does not

**Figure 4:**
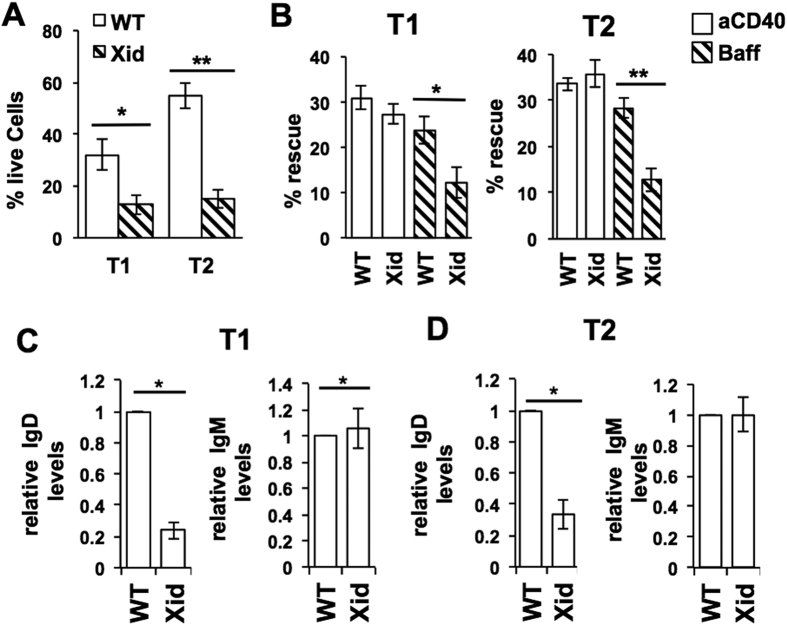
CD40 ligation, but not BAFF, rescues Xid transitional B cells from neglect-induced death. WT or Xid T1 and T2 B cells were purified from spleen and cultured *in vitro* for various periods of time and with various stimuli as indicated. (**A**) Live cell frequencies of T1 or T2 B cells from WT or Xid mice as indicated, cultured in medium alone for 24 h. n = 6; *p < 0.05, **p < 0.0005. (**B**) Purified T1 or T2 B cells from WT or Xid mice as indicated were cultured in medium alone or anti-CD40 mAb (aCD40; 1 μg/ml) or BAFF (Baff; 25 ng/ml) for 24 h, live cell frequencies determined, and the extent of rescue from cell death mediated by aCD40 or BAFF calculated as % Rescue = [{(% dead cells without ligand-% dead cells with ligand)/% dead cells without ligand}*100]. n = 6; *p < 0.05, **p < 0.005. (**C,D**) Splenic T1 (**C**) and T2 (**D**) B cells from WT and Xid mice were additionally stained for IgD together by using cell trace violet dye to label one kind of cells, and relative IgD and IgM levels were measured as MFI values corrected with reference to unstained cells and normalized to WT values in each case as shown. n = 6 mice/group; *p < 0.05.

We next examined the possible role that CD40 ligation plays in ensuring Xid transitional B cell maturation. When purified WT T1 and T2 B cells were cultured without any stimulus, T1 cells showed much more death than T2 cells did (Fig. 4A). However, when T1 and T2 B cells from WT and Xid mice were compared, it was evident that Xid transitional B cells, and T2 B cells in particular, were much more susceptible to death in culture than their WT counterparts (Fig. 4A).

We next tested either BAFF or CD40 ligation (with an activating anti-CD40 monoclonal antibody (mAb) in rescuing T1 and T2 cells from neglect-induced death (Supplementary data Fig. S5A). Since the extent of T1 and T2 B cell death *in vitro* was different between WT and Xid genotypes, the extent of rescue from cell death mediated by BAFF or CD40 ligation was also calculated (see Methods). Both BAFF and CD40 ligation brought about substantive and comparable rescue of WT T1 and T2 B cells (Fig. 4B). However, Xid T1 and T2 B cells showed very poor rescue in response to BAFF, yet CD40 ligation induced their rescue at levels comparable to that of WT cells (Fig. 4B). We tested BAFF over a wide range of concentrations and found that over this range, the extent of rescue mediated in either WT or Xid T 1 B cells was similar (Supplementary data Fig. S5B).

These data suggested that Xid T1 B cells were likely to be different from WT T1 B cells despite their numerical equivalence. Indeed, IgD levels on Xid T1 and T2 B cells were lower than on their WT counterparts, although IgM levels did not differ (Fig. 4C,D).

**Figure 5:**
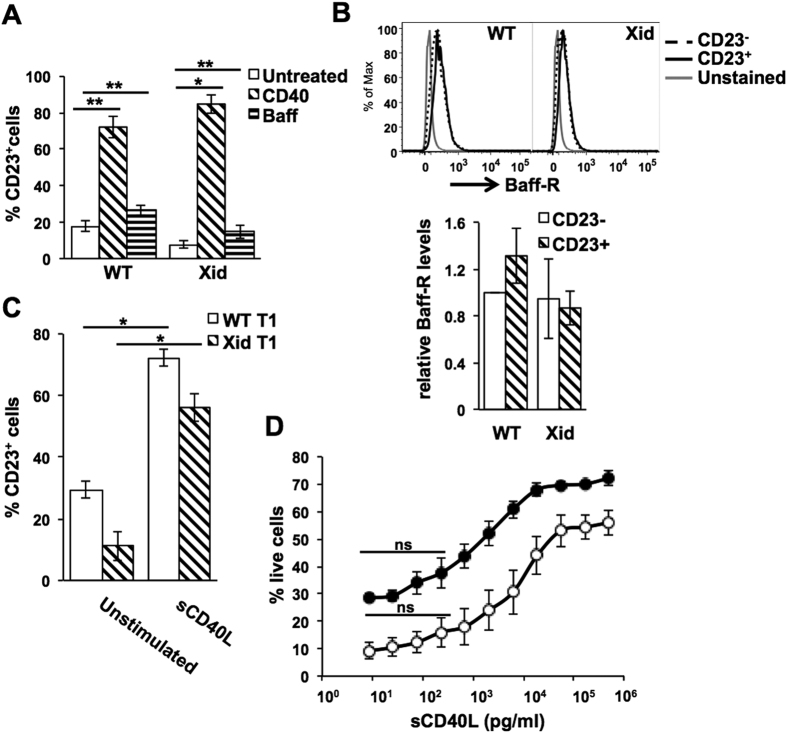
CD40L mediates survival in WT and Xid T1 B cells. (**A**) Purified T1 B cells from WT or Xid mice as indicated were cultured in medium alone or anti-CD40 mAb (aCD40; 1 μg/ml) or BAFF (Baff; 500 ng/ml) for 17 h, and frequencies of CD23-expressing live cells determined. n = 4; *p < 0.05, **p < 0.005. (**B**) BAFF-R levels on CD23 + and CD23- cells from anti-CD40-treated cells from experiments in (**A**) are shown, and BAFF-R MFI values were calculated as normalized to those on CD23- WT T1 B cells. (**C**) Purified T1 B cells from WT or Xid mice as indicated were cultured in medium alone or sCD40L (500 ng/ml) for 17 h, and frequencies of CD23-expressing cells determined. n = 3; *p < 0.005. (**D**) Purified T1 B cells from WT (filled circles) or Xid (open circles) mice as indicated were cultured in medium alone or varying concentrations of sCD40L for 24 h, and live cell frequencies determined. Live cell frequencies in unstimulated T1 WT and Xid B cells were 29.4 ± 2.9% and 11.1 ± 4.7% respectively. n = 3. The sCD40L concentrations at which no significant rescue from cell death was seen are indicated (ns).

CD40 ligation has been shown to induce expression of CD23 on a range of B cell lineage stages^47^, and CD23 is a marker used to discriminate between T1 and T2 B cell stages^48^. We therefore tested to see if either BAFF or CD40 ligation *in vitro* induced CD23 on T1 B cells. Both WT and Xid T1 B cells showed strong induction of CD23 upon CD40 ligation, while BAFF treatment induced relatively less prominent induction of CD23 (Fig. 5A). All concentrations of BAFF tested gave similar very modest induction of CD23 in WT (p < 0.06), though not in Xid T1 B cells (Supplementary data Fig. S5B). However, CD40 ligation-mediated CD23 induction was not accompanied by any increase in the levels of BAFF-R, a characteristic of T2 B cells^49^ (Fig. 5B), indicating that T1 B cells were not undergoing transition to T2 B cells in culture, suggesting that CD40 ligation likely transduces a survival signal.

We also tested if the physiological ligand of CD40, CD40L, could also induce similar rescue of Xid T1 B cells *in vitro*. Recombinant aqueous-phase CD40L (sCD40L) did indeed bring about efficient and equivalent CD23 induction WT and Xid T1 B cells (Fig. 5C). In addition to activated T cells, CD40L is also secreted by platelets^50^, and circulating sCD40L levels of 100–400 pg/ml have been reported^51^,^52^,^53^,^54^. We were unable to detect any serum sCD40L in ELISAs capable of detecting a minimum of ~100 pg/ml. However, when we titrated sCD40L, it was evident that levels below ~300 pg/ml were unable to induce any substantial rescue from cell death in T1 B cells from either WT or Xid mice (Fig. 5D). These data suggest that circulating sCD40L is unlikely to be a major contributor of survival signals to transitional B cells, reinforcing the probable requirement for cell-surface CD40L on T cells for such signalling, although the data presented here do not provide unequivocal evidence that the CD40L signals required necessarily originate from CD4 T cells.

### BAFF-R levels on T1 and T2 B cells from WT and Xid mice

**Figure 6:**
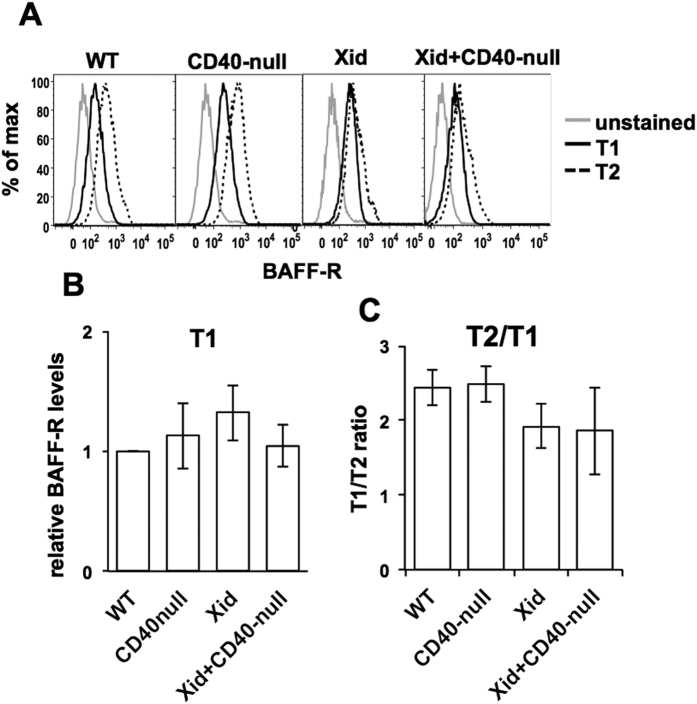
BAFF-R levels on WT, CD40-null, Xid and Xid + CD40-null T1 and T2 B cells. (**A**) BAFF-R levels are shown as histograms on splenic T1 and T2 B cells from various genotypes as indicated were gated as previously described. Unstained splenic T1 B cells were used as controls. (**B**) MFI values of BAFF-R levels on T1 B cells from experiments in (**A**) were calculated as normalized to those on WT T1 B cells. (**C**) The increase of BAFF-R levels on T2 B cells compared to T1 B cells was calculated from the MFI values of BAFF-R levels on T1 and T2 B cells of each genotype from experiments shown in (**A**).

Since Xid transitional B cells were less efficiently rescued from neglect-induced death by BAFF than WT cells were, we tested the BAFF-R levels on these cells in WT, CD40-null, Xid and Xid+ CD40-null double mutant genotypes. BAFF-R levels on T1 B cells were comparable in all four genotypes (Fig. 6A,B). The normal increase of BAFF-R levels from T1 to T2 B cells was quantified and was well detectable in WT and CD40-null mice (Fig. 6B,C). However, BAFF-R upregulation was relatively modest in Xid T2 B cells as well as in Xid+ CD40-null T2 B cells (Fig. 6B,C). Thus, the lack of BAFF-responsiveness in T1 B cells was likely to be independent of BAFF-R levels, while poor induction of BAFF-R levels in the absence of functional Btk on T2 B cells might be a contributory factor in their poor response to BAFF.

### NF-kappaB pathway activity in WT versus Xid T1 and T2 B cells

The T1-T2 maturation defect in Xid transitional B cells, coupled with their poor survival *in vitro* and efficient rescue from death with CD40 ligation, suggested that regulation of cell death may be a major issue. Roles for both the canonical and the non-canonical NF-kappaB pathways have been implicated in the T1-T2 transition process and cell survival^55^,^56^,^57^,^58^. We therefore analyzed these pathways both in *ex-vivo* transitional B cells and in transitional B cells *in vitro* upon BAFF treatment or CD40 ligation.

**Figure 7:**
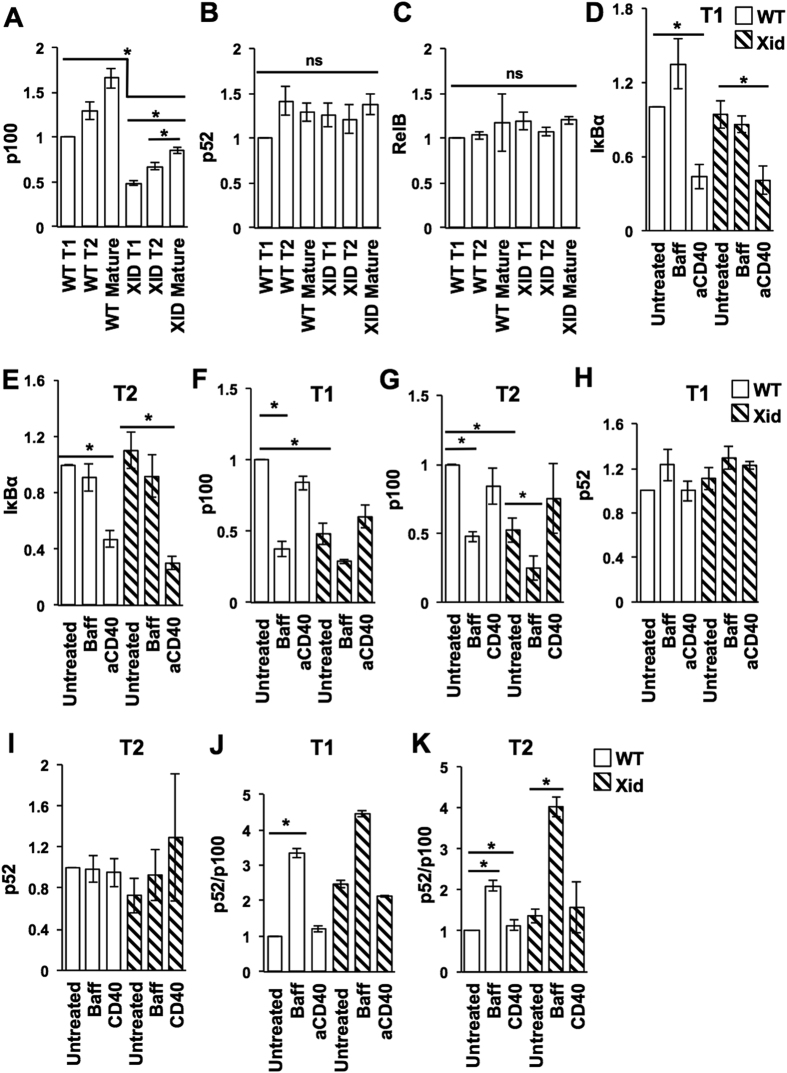
CD40 ligation engages the canonical NF-kappaB pathway in Xid transitional B cells. (**A–C**) T1, T2 or mature (B220+CD93−) splenic B cells were sort-purified from WT or Xid mice, lysed and subjected to Western blot analysis for p100, p52, and RelB proteins as described (see Materials and Methods). Examples of actual blots are shown (Supplementary data Fig. S6). After densitometric analysis, intensities were normalized to WT T1 levels. (**D–K**) T1 or T2 splenic B cells were sort-purified from WT or Xid mice and cultured for 6 h in the presence (or absence; untreated) of either anti-CD40 mAb (CD40/aCD40; 1 μg/ml) or BAFF (Baff; 100 ng/ml), then lysed and subjected to Western blot analysis for I-kappaB-alpha and p100/p52 proteins as described (see Materials and Methods). Examples of actual blots are shown (Supplementary data Fig. S6). After densitometric analysis, intensities were normalized to WT (untreated) levels in each group, and p52/p100 ratios calculated. n = 3 independent experiments, each with cells pooled from 10–12 mice. *p < 0.05.

*Ex vivo*, both transitional and mature B cells from Xid mice showed lower levels of the p100 protein (Fig. 7A,B, Supplementary data Fig. S6). This is consistent with reports that BCR-mediated tonic signals maintain B cell levels of p100, which is the precursor of the effector p52 molecule mediating the non-canonical NF-kappaB pathway^18^,^59^,^60^. Notably, transitional Xid B cells showed even lower p100 levels than mature Xid B cells did (Fig. 7A), suggesting that transitional B cells may be particularly p100-limited. The levels of p52 and RelB were not different between any of the WT and Xid B cell stages *ex vivo* (Fig. 7B,C, Supplementary data Fig. S6).

We next stimulated sort-purified T1 and T2 B cells from WT or Xid mice *in vitro* with BAFF or with anti-CD40 mAb, and measured the loss of IkappaB-alpha 6 h later as an indication of activation of the canonical NF-kappaB pathway. Both WT and Xid T1 and T2 B cells showed efficient and equivalent loss of IkappaB-alpha upon CD40 ligation (Fig. 7D,E, Supplementary data Fig. S6). Unstimulated Xid T1 and T2 B cells showed lower p100 levels than WT cells did after 6 h of culture (Fig. 7F,G). Both WT T1 and T2 B cells showed loss of p100 levels (Fig. 7F,G) and increase in p52/p100 ratios (Fig. 7J,K) upon BAFF treatment, indicating activation of the non-canonical NF-kappaB pathway. CD40 ligation did not show any loss of p100 levels (Fig. 7F,G) or change in the p52/p100 ratios (Fig. 6J,K) in either WT or Xid T1 or T2 B cells, while BAFF did not induce any detectable loss of I-kappaB-alpha in WT or Xid T1 or T2 B cells (Fig. 7D,E). Although both CD40 ligation^61^ and BAFF^62^ have been reported to activate both NF-kappaB pathways, this has not been shown in transitional B cells, which express a different repertoire of BAFF receptors.

**Figure 8:**
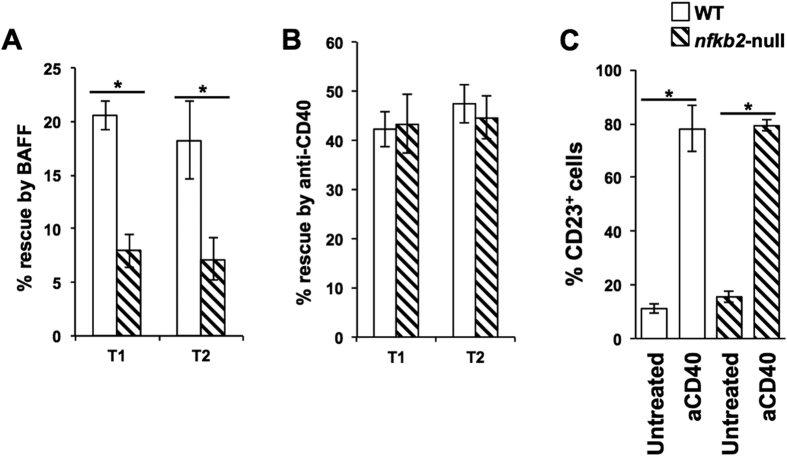
CD40-mediated rescue of T1 and T2 B cells from death is independent of the non-canonical NF-kappaB pathway. (**A,B**) Purified T1 or T2 B cells from WT or *nfkb2*-null mice as indicated were cultured in medium alone or with BAFF (panel A; Baff, 25 ng/ml), or with anti-CD40 mAb (panel B; aCD40, 1 μg/ml) for 24 (T1) or 36 (T2) h, live cell frequencies determined, and the extent of rescue from cell death mediated by BAFF or anti-CD40 calculated. n ≥ 3; *p < 0.05. (**C**) Purified T1 B cells from WT or *nfkb2*-null mice as indicated were cultured in medium alone or anti-CD40 mAb (aCD40; 1 μg/ml) for 17 h, and frequencies of CD23-expressing live cells determined. n = 4; *p < 0.05.

These data suggested the possibility that CD40 ligation-mediated rescue from T1 and T2 B cell death was independent of non-canonical NF-kappaB signalling. We tested this directly by using T1 and T2 B cells sort-purified from p100-null mice. BAFF treatment did not rescue p100-null T1 and T2 B cells from death (Fig. 8A). However, CD40 ligation efficiently rescued p100-null T1 and T2 B cells from death (Fig. 8B) and induced CD23 on T1 cells (Fig. 8C).

## Discussion

Deficiency of functional Btk leads to complex quantitative defects in B cell differentiation. In Btk-deficient humans, B cell differentiation in the bone marrow is substantially affected, to the point that the disease phenotype is X-linked agammaglobulinaemia (XLA)^63^,^64^,^65^. In Xid as well as in Btk-mutant mice carrying the same Btk mutation found in XLA, bone marrow B lymphopoiesis is relatively unaffected^15^,^63^,^66^. One possible contributor for Btk-redundancy in the mouse bone marrow could be the ability of other Tec family kinases such as Tec itself to substitute for Btk, since mice lacking both Btk and Tec show a profound block in B cell development in the bone marrow^21^. However, when Xid bone marrow is simply put into competition with WT bone marrow in mixed bone marrow chimeras, Xid lymphopoiesis is clearly poor^67^,^68^. Even in the periphery, while mature B cell numbers are clearly reduced by the Xid defect, the deficit is relatively modest^69^,^70^. The major Xid deficit is in the T-independent functions of these B cells, explicable by the necessity of Btk for optimal BCR-mediated signalling^3^,^10^,^11^,^69^. However, BCR-mediated signalling is also required for peripheral maturation of B cells through the early transitional stages^7^,^19^ as well as for the survival of mature B cells^60^. Yet, transitional B cell stages appear relatively unaffected in Xid mice^70^, although the defect in mature follicular B cells is quite notable^69^.

These ambiguities in the contributions of Btk to various BCR-mediated signalling outcomes are compounded by two other issues; one, that Btk is a signalling intermediate downstream not only of the BCR but also of other B cell-surface molecules^29^,^71^,^72^, and two, that in addition to the BCR, members of the Tnf-receptor family recognizing BAFF also mediate maturation and survival signals on peripheral B cells^9^,^56^,^57^.

On this background, a plausible interpretation of the data reported in literature that combinations of the Xid genotype with either the nu/nu or the CD40-null genotypes led to severe loss of peripheral but not bone marrow B lineage cells^33^,^34^, was that T cells provide Btk-independent signals for peripheral B cell maturation and survival via CD40 on B cells. Our data provide evidence that this is indeed the case.

In fact, our data indicate that the major numerical deficit in the peripheral B cell compartment in Xid mice lies at the transition from immature to mature follicular B cells whose survival is known to be compromised^20^,^69^, suggesting adequacy of signals providing maturation and/or survival signals until that point, with the caveat that, as B cell turnover rates change, so do the cell maturation rates^73^. However, our data combining the Xid genotype with either the TCRbeta-null or the CD40-null genotypes show that these double-mutant genotypes have a specific reduction of B lineage cell numbers at the T2 (but not the T1) stage, accompanied by a profound loss of subsequent stages of maturation. Thus, the consequence of a combined absence of both functional Btk and T cells and CD40 is not simply an exacerbation of the Xid defect, but an ‘upstream’ shift of the differentiation block to the T1-T2 transition point. The evidence that adding the MHCII-null genotype to the Xid genotype also brings about a similar upstream shift of the B cell maturation defect indicates that CD4 T cells are essential to provide this maturation signal. The bone marrow B lineage in these double-mutant genotypes remains comparable to the Xid genotype, indicating that there are no Btk-independent contributions T cells and/or CD40 make to B lineage development in the bone marrow.

Since transitional B cell maturation is essentially normal in the TCRbeta-null, CD40-null or the MHCII-null genotypes alone, these data indicate that transitional B cell maturation and/or survival can be signalled via two mutually redundant pathways, one Btk-dependent and T cell/CD40-independent, and the other Btk-independent but CD4 T cell/CD40-dependent.

Physiologically, the ligand for CD40, CD40L, is mainly expressed on activated T cells, although there is some expression on other cell types as well^50^,^74^,^75^. Platelets are a major source for detectable serum levels of sCD40L^50^. While levels in the range of 100–400 pg/ml have been reported^51^,^52^,^53^,^54^, we could not detect any levels above 100 pg/ml. In any case, while sCD40L can rescue Xid transitional B cells from cell death that did not happen at levels below ~ 300 pg/ml suggesting a way in which CD40L on activated CD4 T cells may be specifically important for Btk-independent transitional B cell maturation. However, an important caveat must be noted here, namely, that our data do not provide unequivocal evidence that the CD40L signals required necessarily originate from CD4 T cells, in the absence of data using CD4 T cell-specific deletion of CD40L in the Xid genotype.

Since T1 B cell numbers in the double-mutant mice are no higher despite a loss of T2 numbers, it is possible that the T1-T2 block in these double-mutant genotypes is not so much failure of developmental transition but rather of T2 cell survival, although more complex scenarios such as a mild failure of survival of T1 cells and a severe failure of survival in T2 cells, with or without a failure of developmental transition, are also possible. B lineage cells can receive survival signals through BCR and CD40 as well as from BAFF^59^,^76^,^77^,^78^,^79^,^80^, with complex interplay between BCR and BAFF-R of canonical and non-canonical NF-kappaB activation^7^,^42^. Functional BCRs on the B cell surface can trigger Btk-dependent canonical NF-kappaB activation and provide short- lived survival signals to B cells^33^. BAFF regulates many molecular decisions in the B cell lineage^45^,^81^,^82^, particularly in the maturation and survival of transitional stage B cells^81^,^83^. BAFF-R ligation seems to provide more sustained survival signals^33^, possibly involving Btk-independent non-canonical NF-kappaB activation since, in the absence of the non-canonical NF-kappaB family member p100, BAFF-R ligation is unable to provide sufficient survival signals leading to the death of B cells^81^,^84^. However, the BAFF-null B cell defect is more profound than the p100-null B cell defect^85^,^86^,^87^, suggesting that other signalling pathways also probably provide survival signals, such as the PI3-kinase/Akt pathway^80^. CD40 on B cells is known to provide pro-survival signals, possibly via the induction of anti-apoptotic members of the Bcl-2 family^77^, and while Btk has been argued to be involved downstream of CD40 as well^80^, there is also evidence for a Btk-independent TRAF2 recruitment step. Further, both canonical^33^,^77^ and non-canonical pathways^78^,^88^ of NF-kappaB activation have been shown downstream of CD40 signalling.

On this background, our data show that neglect-induced cell death is indeed greater in Xid than in WT transitional B cells, with a more severe survival defect in Xid T2 B cells. BAFF rescued WT but not Xid transitional B cells from this cell death *in vitro*, while CD40 ligation, whether by an agonist mAb or by sCD40L, rescued both WT and Xid transitional B cells equally well. There are subtle qualitative differences reported between T1 and T2 B cells in responding to BCR stimulation; expression of genes encoding anti-apoptotic proteins is induced in T2 and mature B cells and not in T1 cells, possibly through differential regulation of NF-kappaB pathways^7^,^20^. *Ex vivo*, Xid transitional B cells show far lower relative levels of p100 protein compared to WT transitional B cells, although p52 and RelB levels are comparable, indicating that BCR-mediated canonical NF-kappaB-mediated enhancement of p100 levels is hampered in Xid transitional B cells as reported^7^, but there is enough to provide for BAFF-mediated non-canonical NF-kappaB activation to occur well enough to contribute to transitional B cell maturation *in vivo*.

CD40 ligation induces IkappaBalpha degradation in the canonical pathway equivalently in WT and Xid T1 and T2 B cells. In T1 and T2 B cells, BAFF did not detectably induce the canonical NF-kappaB pathway, while CD40 ligation did not detectably induce the non-canonical NF-kappaB pathway. Both CD40 ligation^61^ and BAFF^62^ have been reported to activate both NF-kappaB pathways, but transitional B cells express a different repertoire of BAFF receptors with low TACI levels^89^, which may be responsible for this differential consequence of BAFF signalling in transitional B cells.

However, since CD40 ligation induces rescue from cell death, these data render it plausible that this rescue is independent of BAFF/non-canonical NF-kappaB signals. Indeed, CD40 ligation efficiently rescues p100-null transitional B cells from neglect-induced death *in vitro*. One caveat for this interpretation is that, since uncleaved p100 functions as an inhibitor for non-canonical RelB-dependent NF-kappaB pathway activity, a lack of p100 leads to unrestrained RelB:p50 NF-kappaB activity in Nfkb2−/− cells^90^. However, despite this, BAFF was unable to rescue nfkb2-null T1 cells from neglect-induced death, while CD40 ligation could do so efficiently.

While these data suggested that the canonical NF-kappaB pathway could well play a major role in CD40-mediated transitional B cell survival in the absence of functional Btk, it must be kept in mind that both Btk- and CD40-mediated signals use multiple NF-kappaB-independent pathways as well. This includes signal transduction through PI3K, phospholipase C and PKC, all known to be capable of regulating survival and proliferation of B cells^43^,^45^,^91^,^92^,^93^. The PI3K-Akt signalling axis controls cell growth and metabolism via the mTOR pathway and the AMPK kinase pathway^91^,^92^,^93^. Btk has been shown to control kinetics of Akt, in turn regulating the fine balance between pro-apoptotic and anti-apoptotic activity of Akt under oxidative stress^44^,^94^. Other plausible T cell-derived survival signals for transitional B cells *in vivo* also exist, such as IL-4 from follicular helper T cells that induce glycolysis in B cells through a STAT-6-mediated PI3K-independent pathway and induce Bcl-xL expression^95^. On this background, it was plausible to hypothesize that Xid B cells would show evidence of alterations in cellular metabolism. Interestingly, the data show no such alterations in any of the metabolic correlates measured, either between WT and Xid transitional B lineage cells, or indeed between Xid and double-mutant genotype B cells.

BAFF-R levels are higher on T2 as compared to T1 B cells in WT mice^96^. BAFF-R levels are similar between WT and Xid T1 B cells, yet, BAFF appears to signal less efficiently in Xid T1 B cells. This may be a consequence of the Btk-dependence of BAFF-R-mediated signalling^18^. The induction of BAFF-R on T2 B cells appears to be a consequence of tonic BCR signalling^97^. The increase of BAFF-R levels from T1 to T2 B cells is somewhat lower in Xid mice than in WT mice, consistent with the compromised tonic BCR signalling in Xid T1 B cells, and likely contributing further to the lack of BAFF-mediated signalling in Xid T2 B cells. BAFF binds to other receptors on B lineage cells, namely, TACI and BCMA, and TACI levels have been shown to be lower as well on Xid T1 and T2 B cells^98^ even in comparison to the already low TACI levels on WT T1 and T2 B cells^99^. While TACI has been shown to be redundant in normal mice for transitional B cell maturation^100^, TACI-mediated signals would in any case be poor in Xid transitional B cells, further contributing to the lack of BAFF-mediated signalling in them. BAFF activates the non-canonical NF-kappaB pathway via the BAFF-R, while it activates the canonical NF-kappaB pathway via TACI^101^. The poor induction of either canonical or non-canonical NF-kappaB pathways by BAFF in Xid T1 and T2 B cells may thus have multiple contributory pathways. However, the rescue of neglect-induced death of transitional B cells by CD40 ligation appears to be independent of the non-canonical pathway, since it is unaffected in nfkb2-null cells.

While it is plausible that CD40 engagement, in addition to direct NF-kappaB activation, also provides a rescue pathway for BCR-mediated NF-kappaB activation by rendering it Btk-independent^79^, CD40 signals clearly cannot substitute completely for Btk-dependent BCR and BAFF-R signals, since BAFF-R-deficient Xid mice show a block at the T1-T2 transition despite a normal CD40-CD40L axis^10^. Thus, some baseline BAFF/BAFF-R activity is clearly important for the success of the Btk-independent CD40-mediated transitional B cell rescue pathway we have observed. In fact, our observation that T2 B cells in Xid+ CD40-null double-mutant mice do not show substantial BAFF-R induction may indicate a model in which, in the absence of functional Btk, CD40-mediated signals co-operate with residual tonic BCR signals to induce sufficient BAFF-R expression on T2 B cells to ensure their survival *in vivo*, although we have been unable to detect CD40 ligation-mediated induction of BAFF-R on either WT or Xid T1 B cells *in vitro*.

Thus, our data suggest complex mutually redundant interplays of regulatory pathways during transitional B cell maturation in the periphery, involving both Btk-dependent and Btk-independent components of signalling, and implicate CD4 T cells and CD40L-CD40 as one major Btk-independent component mediated without the involvement of the non-canonical NF-kappaB pathway.

## Methods

### Mice

All mouse strains used, CBA/CaJ, CBA/N (Xid), CD40-null, TCRbeta-null, MHCII-null and Icam1-null (The Jackson Laboratory (Bar Harbor, ME), and *nfkb2*(p100)-null^102^, were bred in the small animal facility of the National Institute of Immunology, New Delhi, India. All mice were used at 6–12 weeks of age. All mice were maintained and used in accordance with the guidelines and with the prior approval of the institutional animal ethics committee of the National Institute of Immunology. All methods were performed in accordance with relevant guidelines and regulations. All experimental protocols were approved by the Institutional Animal Ethics Committee authorized for this purpose.

Given the different background genotypes of the Xid (CBA/CaJ), TCRbeta-null, MHCII-null and ICAM-1-null (C57BL/6), as well as CD40-null (BALB/c) mouse strains, complex but comparable background genotypes were ensured in groups to be compared. For this, Xid female mice were bred with TCRbeta-null, CD40-null, MHCII-null or Icam1-null genotype males, and the resultant F1 progeny were intercrossed to obtain F2 progeny in which male mice of all genotypes, namely, WT, Xid alone, TCRbeta/CD40/MHCII-null alone, and Xid+ TCRbeta/CD40/MHCII-null ‘double-mutant’ (DM) genotypes were generated. Mice of the same age (6–12 weeks) were pooled across litters for analysis.

Genotyping of *tcrb*, cd*40* and *h2aa* (H-2Aalpha) alleles was done on genomic DNA by using primers and PCR conditions described by the Jackson Laboratory (Bar Harbor, ME), and for the Xid mutation by using PCR primers and conditions as described previously^103^. The Icam-1 (CD54)-null status was tested by flow cytometric staining of peripheral blood mononuclear cells with anti-CD54 antibody.

The splenectomy procedure essentially followed a previously described protocol^104^. Briefly, mice were anesthetized using ketamine-xylazine, their left flanks depilated, skin and peritoneum incised, splenic blood vessels pinched and incised to permit spleen removal, and incisions sutured. After ten days post-surgery, mice were gamma-irradiated (5Gy) using a Co60-irradiator (BARC, Mumbai, India), and lymph node B cell lineage analysis carried out 1 month after irradiation.

### Flow cytometry and cell purification

Mice were euthanized, bone marrow, spleen or lymph nodes were isolated, and single-cell suspensions were made after RBC lysis using Gey’s solution if necessary. For analytical flow cytometry, cells were incubated with primary antibodies in staining buffer (1% foetal bovine serum (FBS) and 0.05% sodium azide (Sigma-Aldrich, St. Louis, MO) in phosphate buffered saline (PBS) in the dark on ice for 30 min and then washed, re-suspended, stained similarly with secondary reagents if needed.

The following antibodies were used for flow cytometry; anti-mouse B220-phycoerythrin (PE)-Cy7 (clone RA3–6B2), anti-mouse CD93-allophycocyanin (APC) (clone AA4.1), anti-mouse IgM-peridinin-chlorophyll (PerCP)-eFluor710 (clone II/41), anti-mouse CD23-PE (clone B3B4), anti-mouse CD24-APC-eFluor780 (clone M1/69), anti-mouse CD268 (BAFF-R)-fluorescein isothiocyanate (FITC) (clone eBio 7H22E16), anti-mouse B220-eFluor450 (clone RA3–6B2) and anti-mouse CD21/35-eFluor450 (clone 4E3) (eBioscience, San Diego, CA), anti-mouse CD43-APC (clone S7) and anti-mouse BP1-PE (clone 6C3) (BD Pharmingen, San Jose, CA).

For staining with Mitotracker Green (MG; Molecular Probes, Invitrogen, Eugene, OR), cells were suspended in PBS, dyes added (50 μM), followed by incubation at 37 C for 30 min in the dark. Cells were then washed twice, and then used to stain cell-surface molecules as above.

Detection of reactive oxygen species (ROS) was done by staining cells with 2’,7’-dichlorofluorescin diacetate (DCFDA; 1 μM; Sigma-Aldrich) for 30 min at 37C, followed by washes with serum-free RPMI medium.

Glucose uptake was estimated by incubation in 2-[N-(7-nitrobenz-2-oxa-1,3-diazol-4-yl) amino]-2-deoxy-D-glucose (100 μM; 2-NBDG; Cayman Chemicals, Ann Arbor, MI) for 30 min at 37 C, followed by ice-cold PBS washes.

The rate of protein synthesis was estimated by incubating *ex-vivo* spleen cells in methionine-free medium for 45 min at 37 C, followed by supplementation with 25 μM L-homopropargylglycine (HPG; Molecular Probes, Invitrogen, Eugene, OR) and incubation at 37 C for another 2 h. Cells were then washed with ice-cold PBS with 3% BSA (Sigma-Aldrich) and fixed with 4% paraformaldehyde (Loba Chemie, Mumbai, India) for 10 min at room temperature, followed by washes with PBS containing 3% BSA (Sigma-Aldrich) before incubation for 30 min at 4C in 0.2% Triton X-100 (Sigma-Aldrich) in PBS. The cells were then washed and subjected to the Click-It azide-alkyne detection reaction for 30 min at 37 C. The Click-It reaction components include TRIS (100 mM), L-ascorbic acid (20 mM) (BioBasic, Markham, Canada), CuSO_4_ (1 mM) (Qualigens, Mumbai, India) and Alexa-Fluor488 azide (20 μM) (Molecular Probes).

All stained cell samples were run on a flow cytometer (FACSVerse, FACSCanto, or FACSAria III, Becton and Dickinson, San Jose, CA), and analyzed using FlowJo software (Treestar, San Jose, CA).

For purifying T1 and T2 B cells, single-cell suspensions were stained as above with antibodies to B220, CD93, CD21, CD23 and IgM in RPMI-1640, washed, resuspended in complete medium (RPMI+ 10% FBS) and electronically sorted to purify T1 (B220 + CD93 + IgM + CD23-) and T2 (B220 + CD93 + IgM + CD23 + ) B cells (FACSAria III, Becton and Dickinson). Cell purity and viability were monitored and were routinely > 90%.

### T1 and T2 B cell assays in vitro

Sort-purified T1 or T2 B cells from WT or Xid mice were cultured in RPMI1640 medium with L-glutamine (0.3 mg/ml; Biological Industries), sodium pyruvate (1 mM) and non-essential amino acids (100 mM; Gibco Life Technologies, Carlsbad, CA), 10% heat-inactivated FBS, beta-mercaptoethanol (55 μM; Sigma-Aldrich), penicillin (0.06 mg/ml) and streptomycin (0.1 mg/ml; BioBasic), in the absence or presence of the indicated concentrations of either recombinant human BAFF (Gibco Life Technologies), or of purified tissue-culture-grade anti-CD40 mAb (IC10; eBioscience) or of recombinant soluble CD40L (sCD40L; Peprotech, Rehovot, Israel) for varying periods as indicated. Cell death was estimated flow cytometrically using viability dyes (Sytox Green/Red; Molecular Probes) and acquisition of CD23 expression by staining for CD23. Since the extent of T1 and T2 B cell death *in vitro* was different between WT and Xid genotypes, the extent of rescue from cell death mediated by BAFF or CD40 ligation was also calculated as % Rescue = [{(% dead cells without ligand-% dead cells with ligand)/% dead cells without ligand}*100].

### *Immunoblot analysis*

Sort-purified T1, T2 or CD93-negative mature B cells from WT and XID mice, either *ex vivo* or after 6 -h culture in the presence or absence of anti-CD40 mAb (1 μg/ml) or BAFF (100 ng/ml). Whole cell extracts were prepared and was subjected to immunoblot analyses using anti-NF-kappaB/I-kappaB-alpha antibodies as described earlier^102^. Antibody against p52/p100 (Cat No. 4882) was purchased from Cell Signaling Technology. The gel images were acquired using PhosphorImager (Typhoon 9400, GE Healthcare Life Sciences, Amersham, UK) and ImageQuant 5.2 was used for densitometric quantification of immunoblots.

### Enzyme-linked immunosorbent assays

Serum IgM and IgG levels were assayed by ELISAs. Briefly, 96-well plates were coated with goat anti-mouse Ig (Southern Biotech, Birmingham, AL), followed by serially diluted sera. Bound immunoglobulins were detected using goat anti-mouse IgM/IgG-HRP (Southern Biotech) followed by peroxidase assay using H_2_O_2_ and ortho-phenylene-diamine, with absorbance measured at 490 nm in a micro-plate reader (Tecan, Mannedorf, Switzerland). In some assays, 3,3′,5,5′-Tetramethylbenzidine was used as substrate solution and the absorbance was measured at 450 nm. Concentrations were calculated by extrapolation from appropriate standard curves.

Serum soluble CD40L levels were assayed by a commercial ELISA kit (R&D Systems, Minneapolis, MN).

### Statistical analyses

Student’s ‘t’ test was used to calculate statistical significance of experimental data.

## Additional Information

**How to cite this article**: Tanwar, S. *et al*. Mediation of transitional B cell maturation in the absence of functional Bruton’s tyrosine kinase. *Sci. Rep.* **7**, 46029; doi: 10.1038/srep46029 (2017).

**Publisher's note:** Springer Nature remains neutral with regard to jurisdictional claims in published maps and institutional affiliations.

**Publisher's note:** Springer Nature remains neutral with regard to jurisdictional claims in published maps and institutional affiliations.

## Supplementary Material

Supplementary Figures
