# Supplementary material for: Mediation of transitional B cell maturation in the absence of functional Bruton’s tyrosine kinase
Source: Sci Rep. 2017 Apr 5;7:46029. doi: 10.1038/srep46029 (PMC5380950; doi:10.1038/srep46029)
Supplement: Supplementary Figures [file srep46029-s1.pdf]

Mediation of transitional B cell maturation in the absence of functional Bruton's tyrosine kinase

Shalini Tanwar[1*], Atika Dhar[1*], Vineeth Varanasi[1], Tapas Mukherjee[1], Ramanamurthy Boppana[2], Soumen Basak[1], Vineeta Bal[1†], Anna George[1†], Satyajit Rath[1†‡]

[1]National Institute of Immunology, New Delhi, India; [2]National Centre for Cell Sciences, Pune, India

Supplementary figures

Fig. S1. Gating strategies for B cell lineage stages in bone marrow and spleen

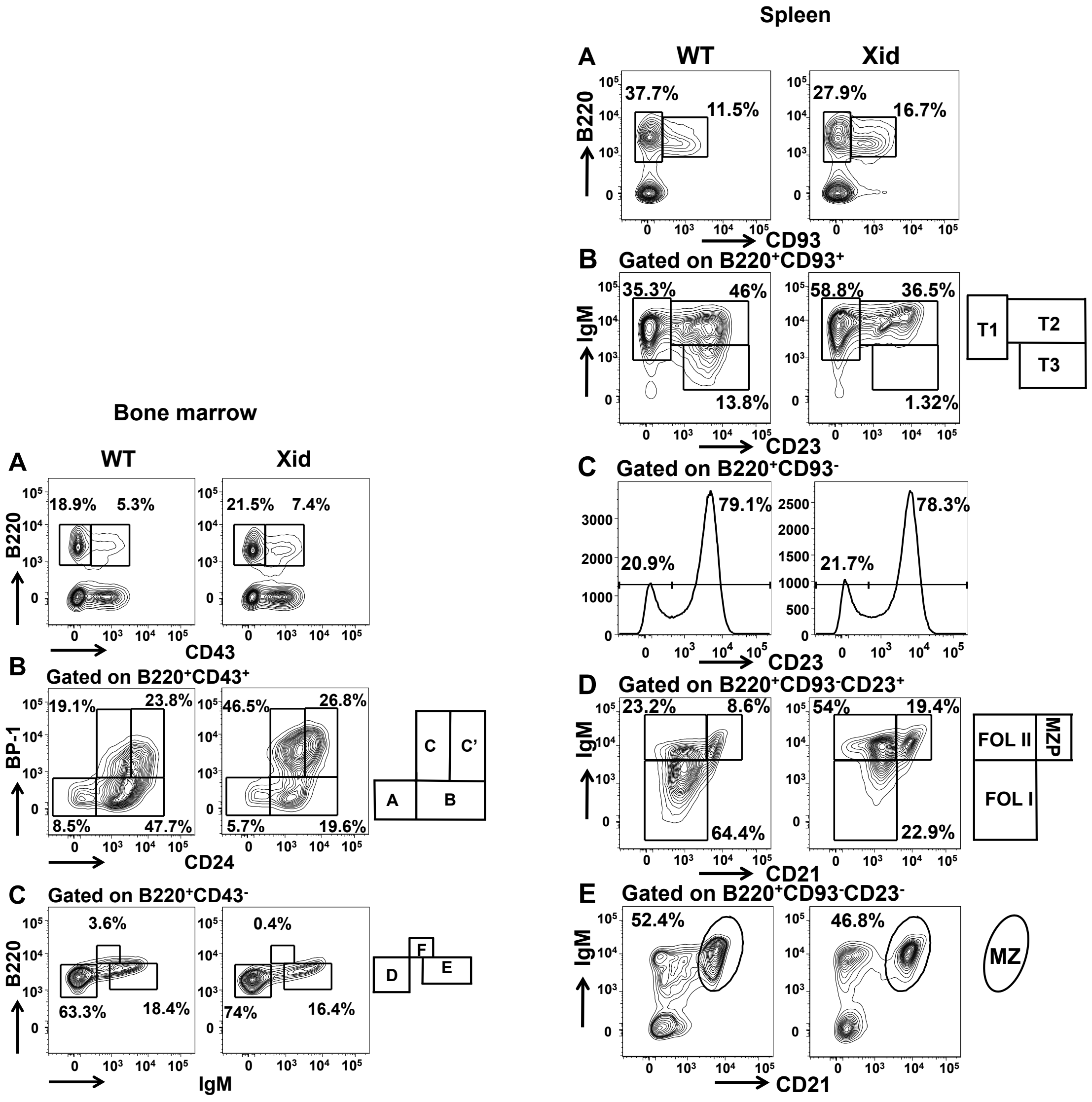

Ex-vivo bone marrow cells from mice of indicated genotypes were stained for B220, CD43, CD24, BP-1 and IgM in order to identify bone marrow B lineage developmental stages, which were gated as indicated following reported nomenclature (ref. 37).

Ex-vivo spleen cells from mice of indicated genotypes were stained, after erythrocyte lysis, for B220, CD93, CD23, CD21 and IgM to identify stages in peripheral B cell maturation and differentiation as shown following reported nomenclature (ref. 39).

Fig. S2. Bone marrow B cell differentiation is only modestly altered in Xid mice, and is not further altered in bone marrow of Xid+CD40/TCRbeta-null mice

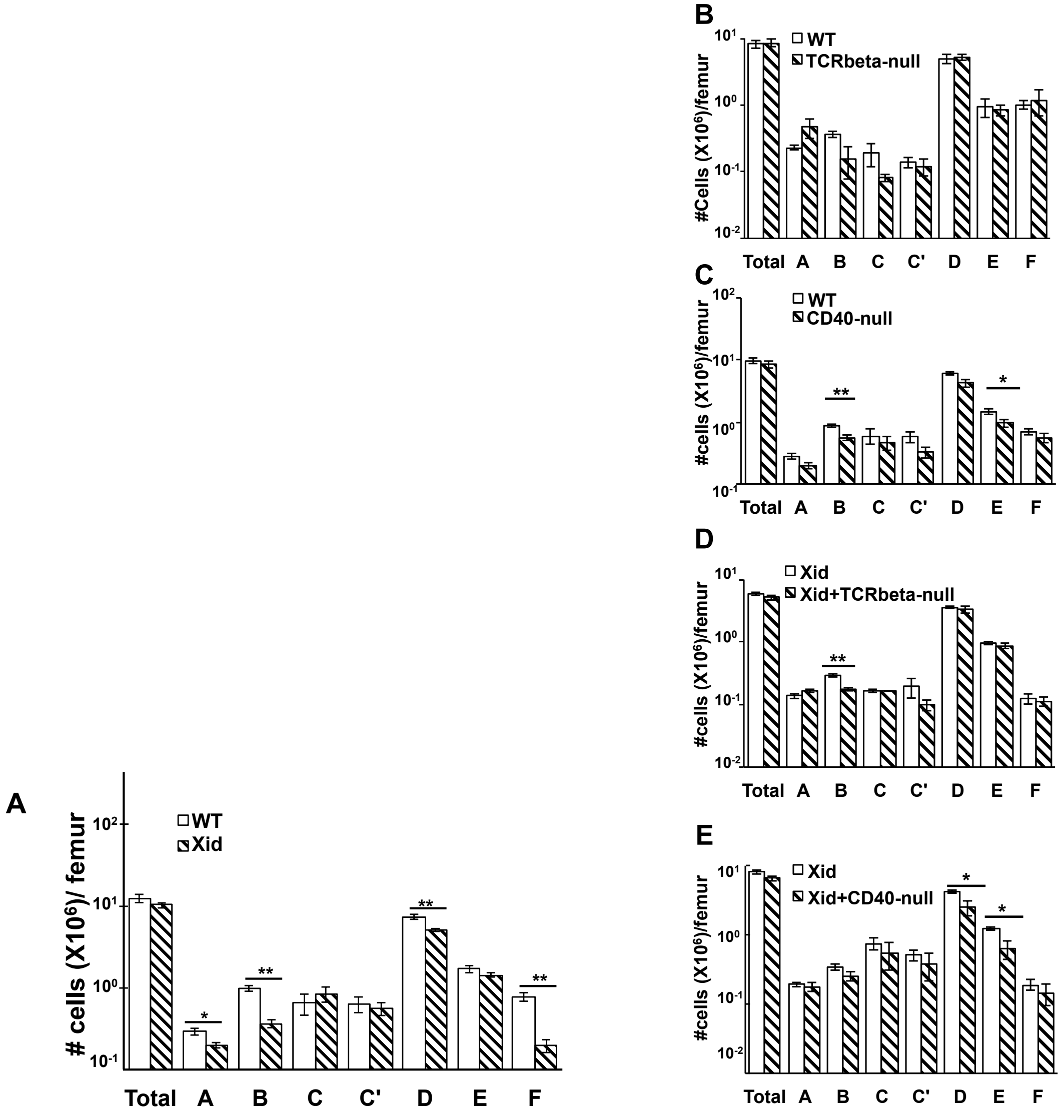

(A) Femoral bone marrow cells from WT and Xid mice were stained for identification of various B lineage cell stages (Fig. S1), and numbers estimated (n=9; *, $p<0.05$; **, $p<0.005$).

(B-E) Femoral bone marrow cells from mice of indicated genotypes were stained for identification of various B lineage cell stages (Fig. S1), and numbers estimated (n=4-6; *, $p<0.05$; **, $p<0.01$)

Fig. S3. **Serum IgG levels in ~8 week-old CD40-null mice**

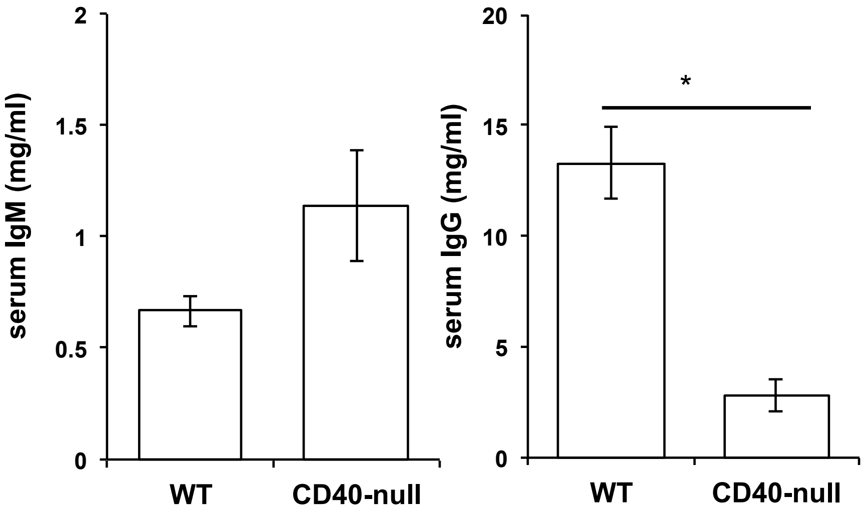

Sera from 8-9 week-old littermate WT or CD40-null mice as shown were analyzed for IgM and IgG levels (n=5).

Fig. S4. Metabolic parameters of T1 and T2 stage B cells are unaltered in Xid mice

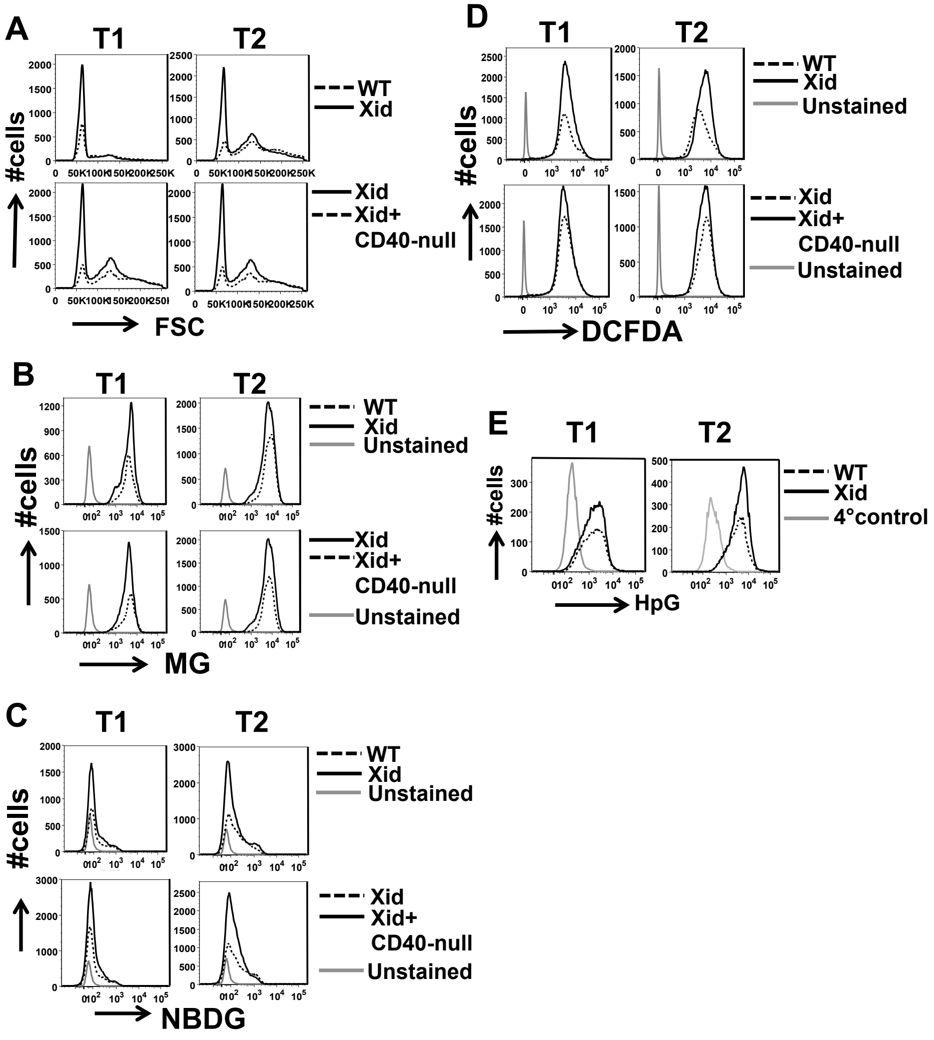

Spleen cells from mice of indicated genotypes were stained as described for the various parameters shown (see Methods).

Fig. S5. CD40 ligation, but not BAFF, efficiently rescues Xid transitional B cells from neglect-induced death

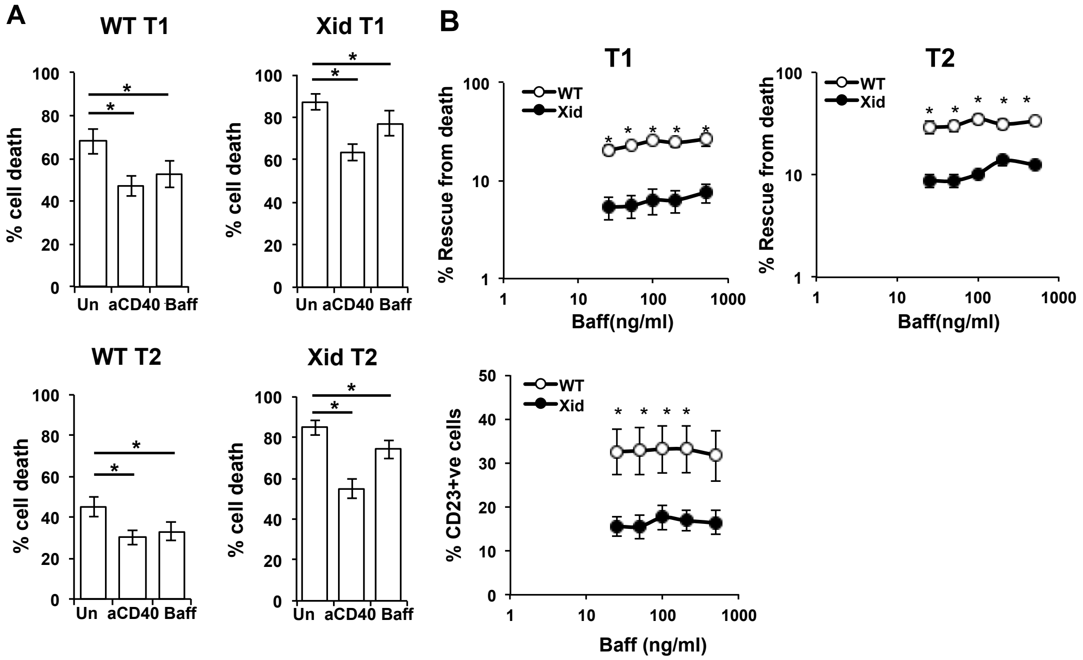

(A) Purified T1 or T2 B cells from WT or Xid mice as indicated were cultured in medium alone (Un) or anti-CD40 mAb (aCD40; 1 µg/ml) or BAFF (25 ng/ml) for 24 h, and dead cell frequencies determined. n=6; * $p<0.05$.

(B) Purified T1 or T2 B cells from WT or Xid mice as indicated were cultured in medium alone or with varying BAFF concentrations as indicated for 24 h, and dead cell frequencies determined. The extent of rescue from cell death (panel B) was calculated as % Rescue =[{(% dead cells without ligand-% dead cells with ligand)/ % dead cells without ligand}*100]. Similarly, purified T1 B cells from WT or Xid mice as indicated were cultured in medium alone or with varying BAFF concentrations as indicated for 17 h, and frequencies of cells expressing CD23 were determined. Background values in the absence of any BAFF were 14.6 $\pm$3.3% and 8.1$\pm$1.3% for WT and Xid T1 B cells respectively. n=4-6, $p<0.05$.

Fig. S6. Levels of NF-kappaB p100 protein are low ex vivo in Xid B cells, and CD40 ligation mediates only canonical NF-kappaB pathway activation in transitional WT and Xid B cells

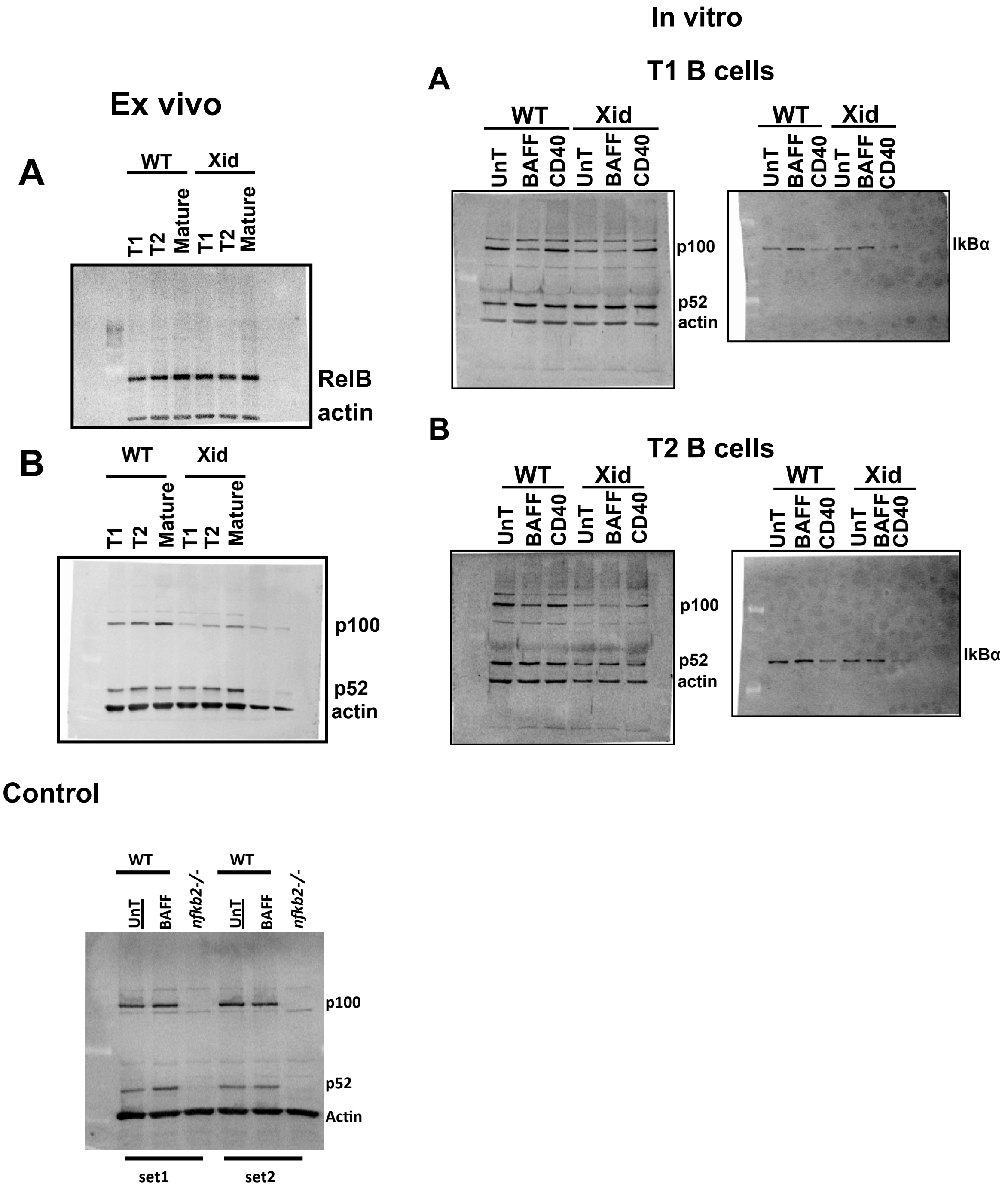

Ex vivo: T1, T2 or mature (B220+CD93-) splenic B cells were sort-purified from WT or Xid mice, lysed and subjected to Western blot analysis for p100, p52, and RelB proteins as described

(see Materials and Methods). The unlabeled lanes in 'B' were from cell lines being tested for expression levels.

In vitro: T1 or T2 splenic B cells were sort-purified from WT or Xid mice and cultured for 6 h in the presence (or absence; untreated (UnT)) of either anti-CD40 mAb (1 μg/ml) or BAFF (100 ng/ml), then lysed and subjected to Western blot analysis for I-kappaB-alpha and p100/p52, proteins as described (see Materials and Methods).

Control: Since multiple bands were visible on some of these Western blot analyses from cultured cells, the identities of the relevant specific p100 and p52 bands were confirmed by ensuring that bands at these specific expected molecular weight distances were seen in wild-type but not in p100-null cell lysates. Western blot data are shown for splenic cells from two different WT or *nfkb2*-null mice, in which the WT cells were cultured with or without BAFF (25 ng/ml) for 6 h. The p100 and p52 bands are seen clearly in WT but not in nfkb2-null cell lanes, as expected.
